# Fidelity and Adherence to a Liquefied Petroleum Gas Stove and Fuel Intervention during Gestation: The Multi-Country Household Air Pollution Intervention Network (HAPIN) Randomized Controlled Trial

**DOI:** 10.3390/ijerph182312592

**Published:** 2021-11-29

**Authors:** Ashlinn K. Quinn, Kendra N. Williams, Lisa M. Thompson, Steven A. Harvey, Ricardo Piedrahita, Jiantong Wang, Casey Quinn, Ajay Pillarisetti, John P. McCracken, Joshua P. Rosenthal, Miles A. Kirby, Anaité Diaz Artiga, Gurusamy Thangavel, Ghislaine Rosa, J. Jaime Miranda, William Checkley, Jennifer L. Peel, Thomas F. Clasen

**Affiliations:** 1 Fogarty International Center, National Institutes of Health, Bethesda, MD 20892, USA; 2 Berkeley Air Monitoring Group, Fort Collins, CO 80524, USA; rpiedrahita@berkeleyair.com; 3 Division of Pulmonary and Critical Care, School of Medicine, Johns Hopkins University, Baltimore, MD 21287, USA; kendra.williams@jhu.edu (K.N.W.); wcheckl1@jhmi.edu (W.C.); 4 Center for Global Non-Communicable Disease Research and Training, Johns Hopkins University, Baltimore, MD 21287, USA; 5 Nell Hodgson Woodruff School of Nursing, Emory University, Atlanta, GA 30322, USA; lisa.thompson@emory.edu; 6 Department of International Health, Johns Hopkins Bloomberg School of Public Health, Baltimore, MD 21205, USA; Steven.Harvey@jhu.edu; 7 Rollins School of Public Health, Emory University, Atlanta, GA 30322, USA; jiantong.wang@emory.edu (J.W.); ajay.p@emory.edu (A.P.); thomas.f.clasen@emory.edu (T.F.C.); 8 Department of Mechanical Engineering, Colorado State University, Fort Collins, CO 80523, USA; Casey.Quinn@colostate.edu; 9 Department of Epidemiology and Biostatistics, Global Health Institute, College of Public Health, University of Georgia, Athens, GA 30606, USA; John.McCracken@uga.edu; 10 Center for Health Studies, Universidad del Valle de Guatemala, Guatemala City 01015, Guatemala; adiaz@ces.uvg.edu.gt; 11 Department of Global Health and Population, Harvard T.H. Chan School of Public Health, Boston, MA 02115, USA; mkirby@hsph.harvard.edu; 12 Department of Environmental Health Engineering, Faculty of Public Health, Sri Ramachandra Institute for Higher Education and Research, Porur, Chennai 600116, India; thangavel@ehe.org.in; 13 Faculty of Infectious and Tropical Diseases, London School of Hygiene and Tropical Medicine, London WC1E 7HT, UK; Ghislaine.Rosa@lshtm.ac.uk; 14 Department of Medicine, School of Medicine, Universidad Peruana Cayetano Heredia, Lima 15102, Peru; jaime.miranda@upch.pe; 15 CRONICAS Center of Excellence in Chronic Diseases, Universidad Peruana Cayetano Heredia, Lima 15074, Peru; 16 Department of Environmental & Radiological Health Sciences, Colorado State University, Fort Collins, CO 80523, USA; Jennifer.Peel@colostate.edu

**Keywords:** cookstoves, LPG, randomized controlled trial, adherence, fidelity, intervention

## Abstract

Background: Clean cookstove interventions can theoretically reduce exposure to household air pollution and benefit health, but this requires near-exclusive use of these types of stoves with the simultaneous disuse of traditional stoves. Previous cookstove trials have reported low adoption of new stoves and/or extensive continued traditional stove use. Methods: The Household Air Pollution Intervention Network (HAPIN) trial randomized 3195 pregnant women in Guatemala, India, Peru, and Rwanda to either a liquefied petroleum gas (LPG) stove and fuel intervention (*n* = 1590) or to a control (*n* = 1605). The intervention consisted of an LPG stove and two initial cylinders of LPG, free fuel refills delivered to the home, and regular behavioral messaging. We assessed intervention fidelity (delivery of the intervention as intended) and adherence (intervention use) through to the end of gestation, as relevant to the first primary health outcome of the trial: infant birth weight. Fidelity and adherence were evaluated using stove and fuel delivery records, questionnaires, visual observations, and temperature-logging stove use monitors (SUMs). Results: 1585 women received the intervention at a median (interquartile range) of 8.0 (5.0–15.0) days post-randomization and had a gestational age of 17.9 (15.4–20.6) weeks. Over 96% reported cooking exclusively with LPG at two follow-up visits during pregnancy. Less than 4% reported ever running out of LPG. Complete abandonment of traditional stove cooking was observed in over 67% of the intervention households. Of the intervention households, 31.4% removed their traditional stoves upon receipt of the intervention; among those who retained traditional stoves, the majority did not use them: traditional stove use was detected via SUMs on a median (interquartile range) of 0.0% (0.0%, 1.6%) of follow-up days (median follow-up = 134 days). Conclusions: The fidelity of the HAPIN intervention, as measured by stove installation, timely ongoing fuel deliveries, and behavioral reinforcement as needed, was high. Exclusive use of the intervention during pregnancy was also high.

## 1. Introduction

Household air pollution (HAP) is one of the most ubiquitous environmental exposures worldwide, particularly in resource-poor settings. HAP is responsible for 2.3 million deaths and 91 million disability-adjusted life years lost each year [1]. Throughout the last decade, many interventions have attempted to mitigate HAP through cleaner biomass-burning stoves or cleaner energy interventions [2,3,4,5]; however, most intervention trials have failed to achieve clean air quality targets until recently [6]. Proposed reasons for failure include the continued mixed use of traditional and intervention stoves (“stove stacking”, [2,7]) and the infiltration of outdoor air pollution [8]. In HAP trials, as in other implementation trials, fidelity (the extent to which an intervention was delivered as intended [9,10]) and adherence (the extent to which it was adopted by participants) are therefore necessary components of trial evaluation that can identify contextual factors that may underlie effectiveness [11].

**Figure 1:**
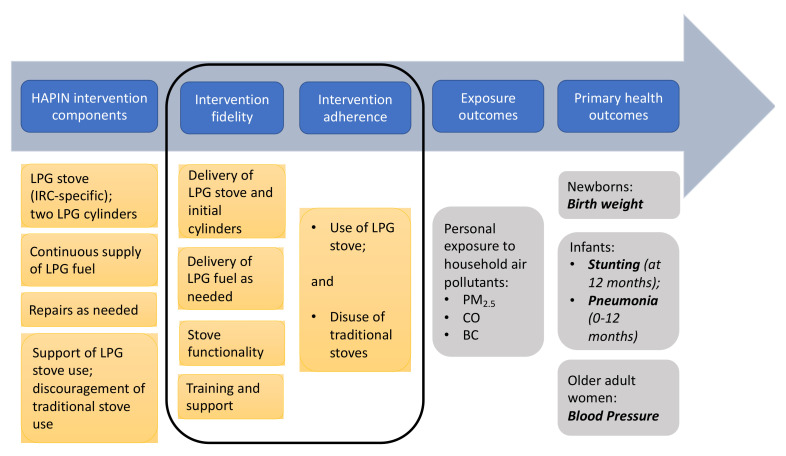
Environmental health theory of change for HAPIN intervention. HAPIN theory of change, outlining intervention components and metrics of fidelity and adherence thought to underlie exposure and primary health outcomes. Yellow boxes represent aspects of the HAPIN trial under investigation in this paper, for the period from enrollment through to the end of gestation (relevant to the first primary outcome: newborn birth weight). Grey boxes represent outcomes further on the change pathway (not reported here). The black outline encircles this manuscript’s areas of focus. PM_2.5_, particulate matter less than 2.5 μm in diameter. CO, carbon monoxide. BC, black carbon.

The Household Air Pollution Intervention Network (HAPIN) trial is a multi-country randomized controlled trial (RCT) of a liquefied petroleum gas (LPG) stove and fuel intervention, complemented with behavioral support, aimed at improving health outcomes among 3195 pregnant women in four countries [12]. Following intervention at between 9 and 20 weeks’ gestation, we are following participants for approximately 5–7 months in pregnancy and an additional 12 months following birth. As of September 2020, all HAPIN births had occurred; the analysis of the first primary outcome, infant birth weight, is underway. Additional primary outcomes to be evaluated after an additional year of follow-up include stunting, severe pneumonia in children under one, and blood pressure in older adult women (Figure 1).

As models suggest that even the occasional use of traditional stoves can lead to exposures above health-relevant guidelines [13], the HAPIN trial was designed to enable and motivate participating households to use their LPG stoves for all their cooking needs, while simultaneously, through the use of behavioral change approaches, urging them to discontinue use of their traditional stoves. To this end, the development of tailored behavioral and stove use reinforcement messages and related media supporting the use of the LPG stove in each research setting was integrated into the intervention and has been described previously [14]. This effort sought to reduce the continued use of traditional stoves to the absolute minimum in intervention homes and to discourage the practice of stove stacking.

Here, we report metrics related to intervention fidelity (stove and fuel delivery in addition to behavioral support) and adherence (stove usage patterns) from intervention through to the first primary endpoint: infant birth weight. The time period of interest here is the period between trial enrollment and the end of the gestation, defined by a live birth, a stillbirth, or study withdrawal prior to birth. Our goal is to understand whether the intervention as delivered was effective in achieving the exclusivity of LPG use during this period, and to report on these key aspects of the intervention process before the main trial outcome (impact on infant birth weight) is known. A similar analysis of intervention fidelity and adherence for the one-year period following birth (relevant to downstream health outcomes) will be conducted at the conclusion of the trial.

## 2. Methods

The design of this evaluation largely followed the recommendations for process evaluation discussed by Moore et al. These include: clarification of the causal assumptions of the intervention, identification of key questions and methods that would shed light on these assumptions, and analysis and reporting of fidelity and adherence data before trial outcomes are known, to avoid potential biases in interpretation [11].

The theory of change underlying the intervention’s desired effect on health outcomes was outlined by the principal investigators and members of the HAPIN Behavioral and Economics Core (Figure 1). Within it, questions related to intervention fidelity and adherence are assumed to be necessary prerequisites to the reductions in exposure that are hypothesized to lead causally to the desired health outcomes, including increased infant birth weight.

### 2.1. Trial Setting

Our trial has been described previously [12,15,16]. We enrolled a total of 3195 pregnant women—approximately 800 in each of four international research centers (IRCs)—in Jalapa, Guatemala; Puno, Peru; Kayonza, Rwanda; and Tamil Nadu, India (Table 1). Gestational age (of 9 to 20 weeks’ gestation per the trial’s eligibility requirements) was determined via ultrasound, and half of the participants were randomized to receive an intervention consisting of a liquefied petroleum gas (LPG) stove, free LPG fuel delivered as needed, free stove repairs, and behavioral reinforcement to support exclusive LPG stove use. Control participants continued using their own (primarily traditional, biomass-burning) stoves for their cooking needs, and are to be compensated for their participation either with an LPG stove at the end of the trial or with other items of their choosing [17].

### 2.2. Intervention Design

LPG stoves and fuel cylinders were different in each research setting, according to local supply and cooking habits, and informed by formative research on cooking practices in each community [14,18]. Each LPG stove had at least two burners; stoves in Guatemala, Peru, and Rwanda had three burners. The stove in Guatemala incorporated a flat griddle or “*comal*” for cooking tortillas (a staple food in the region), while households in Rwanda were supplied with an add-on roasting device to prepare roasted meats. We developed partnerships with local LPG distributors to supply LPG during the trial. LPG cylinder sizes were locally specific (containing, in Guatemala, 11.3 kg of LPG; India, 14.2 kg; Peru, 10 kg; and Rwanda, 15 kg). In all IRCs, each participating household was given at least two LPG cylinders for the duration of the trial, and was instructed to request a refill when the first cylinder was empty. In the research settings in Guatemala, Peru, and Rwanda, research staff were responsible for the initial stove installation and for the delivery and exchange of LPG cylinders. In India, stove installation and LPG cylinder delivery/exchange were conducted directly by a contracted local LPG distribution company, as required by Indian regulations.

Behavioral support of exclusive LPG use (and concurrent discouragement of traditional stove use) was another component of the intervention. Formative research influenced the exact form of these activities in each IRC (see [14]), but across all four IRCs these activities included: (1) a pledge requested of all intervention households at the start of intervention, by which they agreed to use the LPG stove for all their household cooking needs for the duration of the trial; (2) a training session when the intervention was delivered, covering the safety and usage of the LPG equipment; and (3) tailored messages and materials provided at the start of the intervention and thereafter as needed, to encourage and support the exclusive use of LPG and to discourage any continued use of traditional stoves.

Although a large portion of the behavioral support of LPG use (as described above) occurred at the start of the study upon stove installation, additional behavioral reinforcement was provided as needed upon detection of traditional stove use in an intervention household. At these visits, the behavioral reinforcement team spoke with household members about their cooking needs, and a survey was completed to document the participants’ reported reasons for using their traditional stove. The team used the survey responses to lead a discussion with the family about ways to shift future cooking activities to the LPG stove.

### 2.3. Measuring Intervention Fidelity

Intervention fidelity (to what extent the intervention was delivered as intended) was assessed using the following indicators:

*Installation of the intervention package shortly after trial enrollment*: Given that the participants were pregnant women, the timing of the intervention was critical to enable as much coverage by the intervention during gestation as possible. The key metric for this indicator was the time between the visit to randomize the household to intervention or control—conducted in a blinded fashion using a choice of sealed envelopes—and delivery/installation of the stove package (target: < 14 days post-randomization).

Consistent LPG fuel delivery, at a level sufficient to meet all cooking needs, with no gaps in fuel that prompt the use of biomass fuels: Our protocol specified that LPG refills should be delivered within one week of a participant’s request, as the household was instructed to switch to their second cylinder of gas and call to request a refill as soon as their first cylinder ran out. In all IRCs, the fuel cylinder size provided was expected to cover all cooking needs for more than seven days, making it unlikely that participants would deplete their second cylinder while waiting for delivery of a replacement. Here, we calculated the average time between the request and delivery of refill LPG cylinders over gestation (target: < 7 days), as well as the frequency of reports that indicated that a household ran out of LPG fuel and had to revert to cooking with traditional stoves during the gestational period.

*Maintenance and repairs ensuring that the equipment functioned properly and that issues were addressed quickly*: Proper maintenance of the LPG stove and cylinder was checked at every prenatal LPG delivery visit. Participants were encouraged to contact study staff between visits if their stove required maintenance or repair. We tabulated the number of repairs completed and the time between the request and fulfilment of repairs.

*Behavioral support and reinforcement of exclusive LPG use*: Fidelity of behavioral reinforcement was defined as the percentage of households receiving the initial LPG equipment training, the percentage of households who agreed to the LPG stove use pledge, and the percentage of households with documented traditional stove use who received at least one behavioral reinforcement visit during pregnancy.

These household-level metrics were then summarized within each IRC as well as across the total study sample.

### 2.4. Measuring Intervention Adherence

Intervention adherence had two key and interrelated components: *use of the intervention stove* and *disuse of traditional stoves*. These two aspects of stove use were assessed using a combination of questionnaires, stove use monitors, and visual observations by research staff.

First, we collected self-reported stove use data using questionnaires at baseline and at two follow-up visits during pregnancy (at 24–28 and 32–36 weeks’ gestation). Relevant questions were asked about which stoves were used in the previous 24 h.

Quantitative stove use monitoring was conducted using stove use monitors (SUMs) [19]. SUMs were primarily used to assess traditional stove use, and were deployed on all traditional stoves (defined as any stove using biomass fuel (e.g., wood, dung, or charcoal) or kerosene) in intervention households upon installation of the LPG stove. The choice to deploy SUMs preferentially on traditional, versus LPG, stoves was motivated by the understanding that exposure to household air pollution (HAP) from traditional stoves must be nearly eliminated in order to achieve clinically meaningful reductions in exposure [13]. SUMs were left in place continuously through to the end of follow-up, with data downloaded every two weeks. For budgetary reasons SUMs were not routinely installed on LPG stoves, although a convenience subset of ~20% of the intervention households did have SUMs installed on their LPG stoves to inform household air pollution models [15]. Details on algorithms used to detect cooking events using SUMs can be found in the Supplementary Materials. In brief, temperature recordings exceeding thresholds specific to each setting and type of stove (LPG stove vs. traditional stove) and lasting for at least 5 min were used to identify cooking events. Measurements following indications of instrument malfunction (e.g., thermocouple errors) were discarded, and households with less than two continuous weeks of SUM data during pregnancy were excluded from SUM analyses.

Visual observations, made by study staff, were the last component of adherence tracking: at every visit to the household during pregnancy (e.g., to collect anthropometric measurements, biological samples, or exposure data), research staff were asked to observe the kitchen and outdoor cooking spaces and to file a report if there was evidence of current or recent traditional stove use (e.g., a biomass-burning stove currently in use or with ashes/heat, indicating recent use). These observations were intended to catch the use of temporary traditional fires and as a check on SUM data, and would trigger behavioral reinforcement visits to the household. All questionnaire and visual observation data for the trial were recorded in REDCap [20].

Stove use data were aggregated at the daily level, and categorized as days with any detected traditional stove use versus days with none. In households with SUMs, we calculated traditional stove use as a proportion of valid stove-use-monitored days: i.e., the number of days in the household with usage of a traditional stove detected, divided by the total number of days with valid SUMs measurements in that household. As a metric of adherence, we also report the number of households who had less than one day with traditional stove use per 30 days (one month) of observation. If a household destroyed or stored their traditional stove at the start of the trial and visual observations did not indicate subsequent installation/use of another, that household was assumed to have 0 days of traditional stove use over the period of observation.

### 2.5. Stove Use in Control Households

The questionnaires to assess stove use in intervention households in the last 24 h (at baseline, 24–28, and 32–36 weeks’ gestation) were also deployed in control households. These data were analyzed to investigate if there was any uptake of LPG stoves in control households over the follow-up period.

## 3. Results

### 3.1. Study Population and Procedures

Between May 2018 and February 2020, 3195 pregnant women across the four research settings were enrolled in the trial and randomized to either intervention or control groups (Table 1). Five women randomized to the intervention group did not receive the LPG stove because they: left the catchment area (*n* = 1), miscarried between randomization and stove delivery (*n* = 1), or withdrew (*n* = 3). An additional 130 women exited prior to or at delivery of the infant for reasons such as maternal death (*n* = 1), miscarriage/abortion (*n* = 15), moving away (*n* = 19), refusal/withdrawal (*n* = 42), and stillbirth (*n* = 53). The last birth occurred in September 2020.

### 3.2. Intervention Fidelity (Delivery of the Intervention as Intended)

#### 3.2.1. Delivery of Intervention Stove

Among the 1585 pregnant women who received the intervention, the median (Q1, Q3) elapsed time between randomization and the delivery of the intervention was 8 (5, 15) days: for Guatemala it was 9 (5, 15); India, 14 (9, 20); Peru, 5 (2, 7); and Rwanda, 11 (7, 20) (Table 2). Overall, 1156 households (72.9%) had an LPG stove installed within the desired 14-day window after randomization. The median (Q1, Q3) gestational age at the time of stove installation/initial fuel delivery was 17.9 (15.4, 20.6) weeks, and the median time under intervention during pregnancy was 149 days (21 weeks, or approximately 5 months).

#### 3.2.2. LPG Cylinder Refills

**Figure 2:**
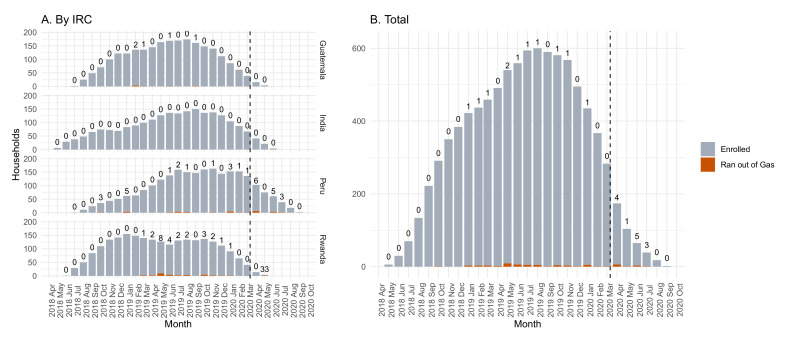
Reported LPG shortages among the enrolled intervention households during pregnancy. (**A**) By IRC; (**B**) total. Grey bars represent the number of HAPIN households being followed-up during pregnancy in each month of the trial and red bars represent number of households that reported having “run out of gas” each month. Numbers above each bar represent the percentage of households who ran out of gas that month among the enrolled intervention households. The dashed line is the start of the COVID-19 pandemic (11 March 2020). Note that in two instances (January 2019 in Peru; March 2019 in Rwanda) a single household reported running out of gas twice that month; all other reports are a single event per household in that month.

Across the trial, there were 10,856 total LPG cylinder refills during gestation. The median (Q1, Q3) time between the request and delivery of LPG cylinders was 1.9 (0.0, 3.3) days across all IRCs, well within the 7-day window specified in the protocol. The number of intervention participants who ever reported that they used their traditional stove because they ran out of LPG during pregnancy was 62 (3.9%): 5 (1.3%) in Guatemala, 0 (0%) in India, 26 (6.6%) in Peru, and 31 (7.9%) in Rwanda. We also provide a figure showing the number of households that reported this issue by month of follow-up (Figure 2). There were no obvious monthly trends in these reports, even considering the start of the COVID-19 pandemic in March 2020: fewer than 5% of total enrolled households reported running out of LPG in any month of follow-up. Notably, 85% of the pregnant women in the intervention households gave birth (or exited the study before birth) prior to the start of the COVID-19 pandemic: *n* = 367 (92%) in Guatemala, 343 (86%) in India, 274 (70%) in Peru, and 362 (92%) in Rwanda.

#### 3.2.3. Repairs

Repairs were conducted on the LPG stove system in 187 households (11.8%) during pregnancy. The wait time in days between the reporting of a stove problem and a successful repair was on average less than one day (Table S2). In most cases, the stove was still usable despite the problem. Often it was the fieldworker who discovered a slight problem upon a household visit to check on the stove.

#### 3.2.4. Stove Use Reinforcement

Of the intervention participants, 100% agreed to the stove use pledge, and pregnant women participated in initial stove use training in 99.1% of the households (Table 2). In the few cases where the pregnant woman was not present, other household members participated in the training; field staff followed-up at subsequent visits to ensure that the pregnant woman was comfortable using the LPG stove.

**Figure 3:**
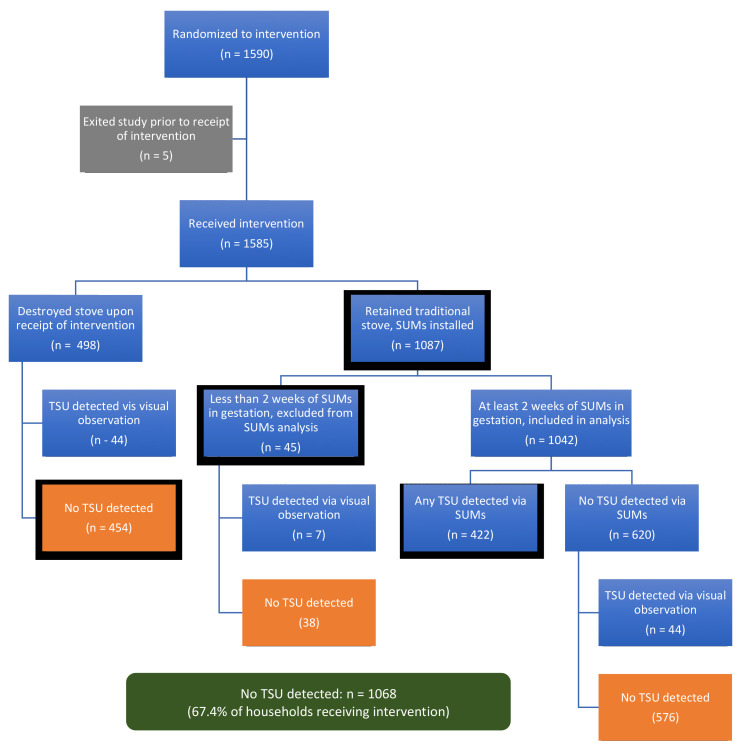
Traditional stove use (TSU) in the intervention households during pregnancy.

A total of 351 intervention households were visited for follow-up behavioral reinforcement: *n* = 52 in Guatemala, 3 in India, 175 in Peru, and 121 in Rwanda (Table 2). This represents 68% of the 517 participants (33% of the intervention arm) who ever had traditional stove use flagged by SUMs or direct observation during pregnancy (Table 3 and Figure 3).

### 3.3. Intervention Adherence (Stove Use)

**Figure 4:**
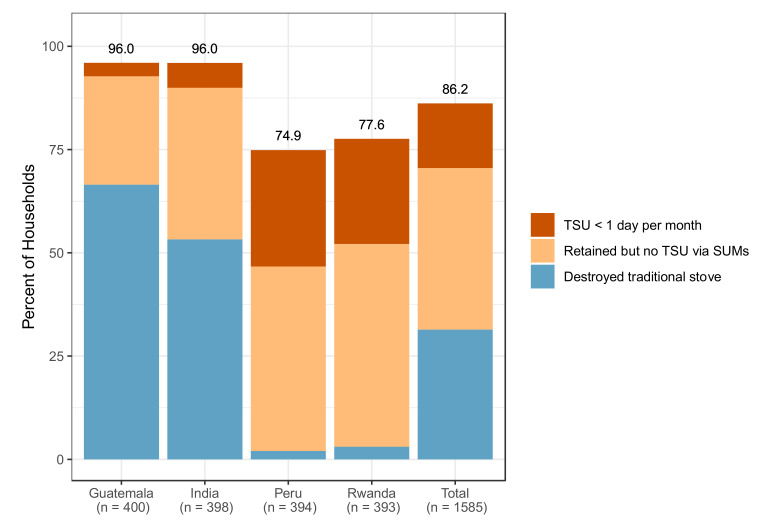
Disuse of traditional stoves in the intervention households. Each stacked bar represents the percent of the intervention households in an IRC who either: destroyed their traditional stove upon LPG installation (blue); retained their traditional stove but no evidence of use was observed over the gestational period (using SUMs, sepia); or used their traditional stove on less than one occasion per month over the gestational period (using SUMs, red). Numbers on top of each bar represent the sum of these categories in each IRC. The total bar presents the average across all four IRCs.

We provide a summary of traditional stove use in the intervention households across the trial in Figure 3, and by IRC in Table 3. As noted above, the primary use of SUMs was to monitor traditional stove use following LPG stove installation in the intervention households. Although the intervention households were not asked to remove or alter their traditional stoves in any way, a substantial proportion of them, particularly in Guatemala (*n* = 266, 66.5%) and India (*n* = 212, 53.2%), destroyed or stored their traditional stove(s) upon installation of the LPG stove. A smaller number of households in Peru (*n* = 8, 2.0%) and Rwanda (*n* = 12, 3.1%) also destroyed or stored their traditional stoves. Of the remaining 1087 households that retained their traditional stoves, SUM data met validity criteria for 1042 (99.1%); coverage of stove-use-monitoring over pregnancy was high (median (Q1, Q3) coverage: 100% (73.3%, 100%) of gestational follow-up time). In over half (59.5%) of these households, no traditional stove use was detected during the monitoring period. Of all the households, 86.2% either destroyed their traditional stove, retained the stove but did not use it, or used it infrequently (< 1 day per month, Figure 4).

Visual observations at household visits were used as a check on SUM data and to identify newly built or installed traditional stoves (to detect the use of stoves as yet unequipped with SUMs). Our data suggest that the existence of unobserved traditional stoves was rare: over the course of participant follow-up during pregnancy, fieldworkers recorded over 16,000 visual observations in households, and among these only 122 (0.8%) indicated a recently used traditional stove with no SUMs installed. Altogether, the complete abandonment of traditional stoves (no traditional stove use detected via SUMs or visual observations) was observed among 67.4% of the households (Table 3 and Figure 3).

**Figure 5:**
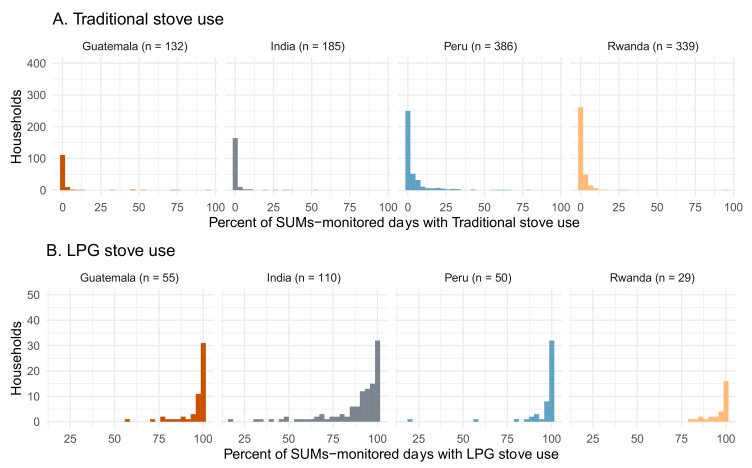
Intervention households’ stove use in gestation as measured by SUMs. (**A**) Percent of stove-use-monitored days with traditional stove use detected via SUMs in the intervention households during pregnancy. Note that protocol called for all the intervention households (~400 in each IRC) to be followed with SUMs installed on traditional stoves, but many households (particularly in Guatemala and India) destroyed their traditional stoves and thus SUMs could not be installed. (**B**) Percent of stove-use-monitored days with LPG stove use detected via SUMs in the intervention households during pregnancy. By design, SUMs were only installed on LPG stoves in a subset of homes.

A small fraction of the intervention households reported needs that resulted in the use of traditional stoves (Table 4). Among them, reasons included the need to cook large quantities of food, to prepare particular dishes, other household members using the traditional stove, issues with LPG supply, or concerns about its safety. The histograms in Figure 5A present data using SUMs, demonstrating that very few of the intervention households in any IRC used their traditional stoves on more than 10% of monitored days. In the households where SUMs detected traditional stove use, the cooking time per day on traditional stoves was, on average, 91 min (approximately 1.5 h, Table 3).

SUM data for LPG stoves during the gestational period were available in a convenience sample of 244 (15.4%) of the intervention households: 55 (13.8%) in Guatemala, 110 (27.6%) in India, 50 (12.7%) in Peru, and 29 (7.4%) in Rwanda (Table 5 and Figure 5B). In these homes, LPG stove use was detected on 98.3% of the monitored days, with a median of 99% of days in Guatemala, 93.8% in India, 99.3% in Peru, and 98.3% in Rwanda. The median time spent using the LPG stove by household was 232 min (over 3.8 h) a day (Guatemala, 299 min; India, 197; Peru, 285; and Rwanda, 231).

**Figure 6:**
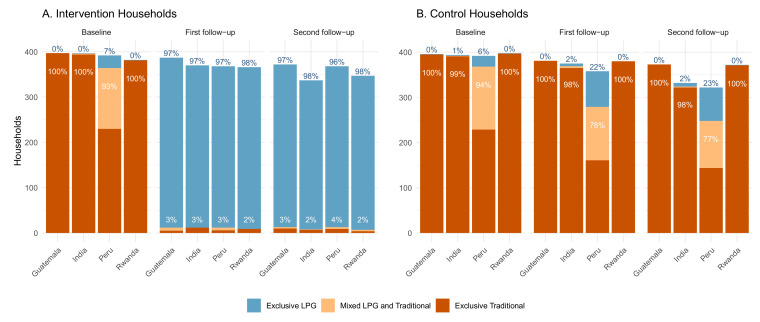
Self-reported stove use. Self-reported stove use in the last 24 h by intervention (**A**) and control (**B**) households at baseline, 1st prenatal exposure monitoring visit (24–28 weeks gestation), and 2nd prenatal exposure monitoring visit (32–36 weeks gestation). Percentages in white are the percent of households, out of the total reporting, that reported any traditional stove use in the previous 24 h (exclusively traditional and/or mixed LPG and traditional). Percentages in blue are the percent of households that reported exclusively LPG use in the previous 24 h.

Finally, stove use data collected by a questionnaire for the intervention households (Figure 6A) demonstrate a shift in self-reported stove use behavior between the baseline and post-intervention visits. Between 93% and 100% of the intervention households reported traditional stove use in the previous 24 h at baseline. At the first follow-up visit (24–28 weeks gestation), less than 4% of the intervention households in any IRC reported using traditional stoves over the previous 24 h. These levels remained largely unchanged at the second visit (32–36 weeks gestation). Meanwhile, between 96% and 98% of the intervention households at each of the four sites reported exclusive use of the LPG stove in the previous 24 h at both post-intervention visits.

### 3.4. Stove Use in Control Arm

The primary means of assessing stove use in the control arm was by self-reporting. As in the intervention arm, between 94% and 100% of participants randomized to the control arm in each IRC reported use of a traditional stove in the last 24 h at baseline (Figure 6B). In Guatemala, India, and Rwanda, there was virtually no reported LPG use in control households during pregnancy (under 2% reporting exclusive LPG use, and very little stacking with traditional stoves and LPG, whether at baseline or follow-up).

Self-reported LPG use among controls was higher in Peru compared to the other IRCs: 6% of control households reported exclusively using an LPG stove in the past 24 h at the baseline visit, 22% at the first pregnancy follow-up visit, and 23% at the second follow-up visit. Although substantial, these numbers are still much smaller than the proportion of the intervention households in Peru that reported exclusive LPG use at follow-up (96–97%) (Figure 6A).

## 4. Discussion

Answering our trial’s fundamental study question—the effect of a clean fuel intervention on human health—requires successful intervention fidelity and adherence. These are prerequisites in the theory of change (Figure 1) to the potential for achieving the exposure reductions believed to be necessary for health gains. In the HAPIN study, an intervention for pregnant women consisting of a multi-burner LPG stove, two cylinders of LPG fuel, and continuous free home delivery of LPG—combined with adequate training and support—can result in a near-complete transition of cooking practices from traditional stoves to LPG during the latter half of gestation: a finding that, to our knowledge, has not been previously reported. Specifically, LPG shortages were reported by less than 4% of the intervention households over the duration of pregnancy. Over 96% of the intervention households reported exclusive use of LPG for cooking at each pregnancy follow-up visit. Delivery of LPG across trial participants was robust, and LPG stoves were well-constructed, with repairs seldom needed. We report a complete transition to exclusive cooking with LPG during pregnancy among over two-thirds of our intervention participants, and minimal stacking with traditional stoves among the others. In over 86% of the intervention households, traditional stoves were either not used at all or used less than 1 day per month of follow-up. These findings were upheld across four distinct study settings, speaking to the generalizability of this specific intervention to different locations with varying cooking practices and cultural contexts.

We report several successes related to intervention fidelity and adherence: first, we succeeded in securing consistent access to the intervention among the study participants, by making the fuel free and delivering it directly to participants’ homes. This access continued despite disruptions associated with the COVID-19 global pandemic. Upon receiving permission from local authorities and following strict COVID-19 prevention and disease control protocols [21], we were able to continue to provide fuel with little interruption, largely because the delivery of household cooking fuel was considered an essential activity in each of our four study regions. We also succeeded in enabling near-exclusive use of the LPG stoves and simultaneous disuse of traditional stoves among the intervention arm participants.

These results are in sharp contrast to previous trials of improved biomass cookstoves, where continued use of traditional stoves was common. In Michoacán, Mexico, for example, adherence to an improved biomass stove intervention was only 50% [22]. In Malawi, use of an improved biomass intervention stove declined over time, with a quarter of the trial households abandoning the intervention stove after one year [2]. By contrast, less stacking with traditional stoves has been reported in previous trials that, similar to ours, intervened with LPG stoves, and where both stoves and fuel were available at no cost to the participants [6,8].

The effectiveness of an environmental intervention to reduce HAP at the household level in low- and middle-income country settings requires that three conditions are met: the intervention must be able to meaningfully reduce important exposures (efficacy); it must reach populations with high levels of exposure to HAP (fidelity); and it must be taken up and used consistently, if not exclusively (adherence). Until recently, efforts to reduce HAP largely failed to meet the first condition, as improved biomass cookstoves were unable to achieve necessary targets in indoor air quality [2,23,24,25]. However, a shift of focus within the sector from improved biomass cookstoves to cleaner fuels such as LPG and electricity, which have been shown to more consistently reduce HAP exposure to levels below WHO guidelines [26], has been a solution to the efficacy issue. Furthermore, the acceptance of LPG as an exclusive cooking technology in different settings suggests that cooks in a diversity of settings accept and even embrace this technology, finding it well-suited to meet a variety of cooking tasks. LPG is regarded by many as a fast, convenient, and cleaner way to cook (e.g., [27,28,29,30,31]). When efforts have been made at an appropriate scale to transition large populations to LPG for cooking, they have often been successful (e.g., [32,33,34]), as long as financial barriers to adoption were removed. Notably, however, stacking with traditional fuels remains extremely common in the context of large-scale governmental efforts to promote LPG for cooking [35].

The primary challenges for clean fuel uptake are related to fidelity and adherence, as the adoption and sustained use of clean fuels at scale faces a number of daunting obstacles. A robust supply chain of LPG fuel must be reliably available at a cost that is affordable to end users [36], and demand must exist to support the supply. LPG cylinders are heavy, even when empty, and newly filled cylinders must regularly reach the household to replace depleted ones. Using an LPG stove requires learning new behaviors, such as lighting the stove, regulating the flame, and checking for safety. Stoves must withstand repeat use, and repairs or replacement must be readily and easily available. Finally, the achievement of health goals will necessitate not only adoption of the new technology, but the simultaneous disadoption of older, customary cooking habits, as even an occasional reversion to traditional stoves can result in excessive exposure to HAP [13]. For the purposes of HAPIN, we planned and piloted both intervention and behavior change communication strategies, including the provision of the stove and fuel intervention free of charge. We worked with local stove manufacturers and LPG companies who provided invaluable experience, advice, and training to trial staff. We assumed responsibility for ensuring consistent stove and cylinder deliveries and repairs, including the provision of two cylinders to each home for easy switching when one cylinder was depleted. We monitored stove use continuously and provided behavioral messaging when traditional stove use was detected by either observation or self-reporting during home visits or by SUMs [14]. We believe the high level of exclusive LPG use observed during pregnancy in this trial to be due to the combination of several factors, namely the acceptability/likeability of the LPG stove and fuel system by the participants; the fact that its provision was free; and the investments of time and labor by our trial staff in training, supporting, and reinforcing exclusive LPG use throughout the trial.

As a result, the study is well-positioned to achieve the reductions in exposure that are thought to be necessary to effect measurable change in birth weight [37].

However, the transferability of this success should not be assumed under conditions where stoves and fuel are accessed through conventional supply chains and where consistent use requires that they be affordable, available, and prioritized by low-income households. Achieving the levels of adoption and use that we observed will almost certainly require government initiatives to further promote access, especially to households well-beyond the proverbial “last mile” [38,39]. Clean fuel programs will also likely require subsidies to reduce the cost of fuel for populations at the base of the economic pyramid [33]. If the intervention achieves significant health gains, some have argued these costs could potentially be offset by savings to the health system [40]. Limiting an intervention to gestation and the first year of a child’s life will reduce its implementation cost, but it is unclear how that may impact future patterns of clean cooking. Still, our study provides compelling evidence that a targeted intervention can be successful in reaching a vulnerable population and that they will willingly accept it, in a manner that has the potential to achieve durable gains in health and development.

There were some potential shortcomings in our study. While we employed multiple complementary strategies to detect traditional stove use in the intervention households, occasional traditional stove use events may have eluded detection. For example, participants and/or their families may have constructed temporary—and undetected—open fires outside the household to cook for large events or to burn trash, and SUM algorithms may have occasionally missed traditional stove use events despite their careful calibration. We feel confident, nonetheless, that the multiple checks put in place to detect traditional stove use (SUMs downloading/placement checking every two weeks; visual observations whenever possible) were successful in capturing the vast majority of traditional stove use events, and that any occasional missed events would not impact our overall findings on adherence.

Another limitation is that, given our emphasis on the detection of traditional stove use events, the bulk of the trial’s stove use monitors were deployed on traditional stoves in the intervention households. We therefore are unable to provide a complete picture of stove use—across LPG and traditional stoves—for more than a small subset of enrolled homes. While this strategy was well-suited to meeting the needs of the trial, it does limit complex analyses of stove use behavior (e.g., of daily or meal-by-meal patterns of cooking across multiple types of stoves) to the subset of homes where LPG stoves, as well as traditional ones, were monitored with SUMs. Despite this limitation, we feel that the results provided here present the most comprehensive data to date on the successful adoption and sustained exclusive use of an interventional cookstove during the prenatal period, as relevant to exposure in utero and infant birth weight. After the conclusion of the trial, a future publication will focus on fidelity and adherence to the intervention during the year of follow-up post infant birth, as these are relevant to postnatal exposures and downstream health outcomes. These data will be interesting to compare to the ones reported here as the intervention will have been in homes for longer periods of time, potentially affecting adherence, repair requirements, and other issues related to sustained use.

## 5. Conclusions

In the context of a randomized controlled trial of an LPG stove and fuel intervention with behavioral reinforcement in four LMIC settings, we report high adoption of the intervention, with the majority of households randomized to the intervention completely eliminating the use of traditional stoves during pregnancy.

## Figures and Tables

**Table 1:** Participants in HAPIN throughout gestation.

Treatment Group	Control				Intervention				Total
IRC	Guatemala	India	Peru	Rwanda	Guatemala	India	Peru	Rwanda	
Enrolled women (N)	400	399	402	404	400	400	396	394	3195
Exits before birth (N)	14	13	44	10	16	12	11	15	135
Live births (N)	386	386	358	394	384	388	385	379	3060
Days in study during pregnancy (randomization to the end of gestation): median (Q1, Q3)	164.0 (144.8, 181.0)	156.0 (136.5, 172.5)	160.0 (136.0, 180.0)	171.0 (154.0, 185.0)	162.0 (143.0, 178.0)	155.0 (136.0, 171.0)	158.0 (138.0, 180.0)	166.0 (152.0, 182.0)	162.0 (142.0, 179.0)
Household size: median (Q1, Q3)	4 (3, 6)	3 (3, 5)	4 (3, 6)	3 (3, 4)	5 (3, 7)	3.5 (3, 5)	4 (3, 5)	3 (2, 4)	4 (3, 5)

**Table 2:** Intervention fidelity.

IRC	Guatemala	India	Peru	Rwanda	Total
Households receiving intervention ^1^	400	398	394	393	1585
Stove and Initial LPG Cylinder Delivery					
Days between randomization and stove/cylinder delivery: median (Q1, Q3)	9.0 (5.0, 15.0)	14.0 (9.0, 20.0)	5.0 (2.0 7.0)	11.0 (7.0, 20.0)	8.0 (5.0, 15.0)
Intervention delivery within 14 days of randomization: N (%)	286 (71.5%)	218 (54.8%)	393 (99.7%)	259 (65.9%)	1156 (72.9%)
Gestational age at start of intervention (weeks): median (Q1, Q3)	17.5 (15.4, 20.6)	18.7 (16.4, 21.7)	17.4 (14.6, 20.1)	18.0 (15.7, 20.4)	17.9 (15.4, 20.6)
Days under intervention during pregnancy (installation date to the end of gestation): median (Q1, Q3)	150.0 (131.0, 167.0)	139.0 (120.0, 157.0)	153.5 (134.0, 176.0)	153.0 (135.0, 170.0)	149.0 (130.0, 168.0)
LPG Refill Delivery					
Time between request and delivery (days) ^2^: median (Q1, Q3)	2.2 (1.9, 2.6)	5.0 (3.0, 7.5)	0.0 (0.0, 0.0)	1.3 (0.5, 3.3)	1.9 (0.0, 3.3)
Stove Use Reinforcement					
Participants who agreed to the stove use pledge: N (%)	400 (100%)	398 (100%)	394 (100%)	393 (100%)	1585 (100%)
Participants who received LPG stove training: N (%)	399 (99.8%)	388 (97.5%)	392 (99.5%)	392 (99.8%)	1571 (99.1%)
Participants with traditional stove use (TSU) who received a behavioral reinforcement visit: N (% of those with TSU)	52 (57.1%)	3 (7.7%)	175 (79.9%)	121 (71.6%)	351 (67.8%)

^1^ A total of *n* = 5 households randomized to intervention (2 in India, 2 in Peru, and 1 in Rwanda) exited the study after randomization and before LPG stove delivery.^2^ Data on delays in timely LPG delivery assume that participants requested LPG by phone prior to fieldworker visits. In some cases, participants did not call for LPG deliveries in advance of bi-weekly fieldworker visits, yielding an interval of 0 days between request and refill (most common in Peru). Given the low reporting rate of running out of LPG (Figure 2), any lack of prior refill requests does not appear to have substantially affected the continuity of LPG supply.

**Table 3:** Traditional stove use (TSU) in the intervention households during pregnancy.

IRC	Guatemala	India	Peru	Rwanda	Total
Households receiving intervention	400	398	394	393	1585
**Households who Destroyed Traditional Stoves (no SUMs)**					
Households who destroyed/stored traditional stove upon intervention: N (%)	266 (66.5%)	212 (53.3%)	8 (2.0%)	12 (3.1%)	498 (31.4%)
Households with subsequent positive visual ID of TSU: N (% of those who destroyed)	43 (16.2%)	0 (0.0%)	1 (12.5%)	0 (0.0%)	44 (8.8%)
**Households who Retained Traditional Stoves (SUMs Installed)**					
Households retaining a traditional stove: N (%)	134 (33.5%)	186 (46.7%)	386 (98.0%)	381 (96.9%)	1087 (68.6%)
Households with valid SUM data (≥ 2 weeks during gestation): N (% of those retaining a traditional stove)	132 (99.2%)	185 (100.0%)	386 (100.0%)	339 (97.7%)	1042 (99.1%)
Days of stove-use-monitoring per household: median (Q1, Q3)	99.0 (29.8, 146.0)	127.0 (92.0, 148.0)	144.5 (121.2, 170.0)	133.0 (92.5, 160.0)	134.0 (97.0, 160.0)
Proportion of gestational follow-up time monitored by SUMs: Median (Q1, Q3)	69.3 (21.6, 100.0)	100.0 (81.5, 100.0)	100.0 (97.9, 100.0)	100.0 (67.6, 100.0)	100.0 (73.3, 100.0)
Percent of stove-use-monitored days with TSU detected: Median (Q1, Q3)	0.0 (0.0, 0.0)	0.0 (0.0, 0.0)	0.6 (0.0, 3.6)	0.0 (0.0, 1.4)	0.0 (0.0, 1.6)
Households with no SUM-detected TSU during pregnancy: N (%) ^1^	105 (79.5%)	146 (78.9%)	176 (45.6%)	193 (56.9%)	620 (59.5%)
Households with < 1 day with TSU per 30 days of monitoring: N (%) ^1^	118 (89.4%)	170 (91.9%)	287 (74.4%)	293 (86.4%)	868 (83.3%)
Traditional stove cooking minutes per day, among those with ≥ 1 day of TSU in gestation: median (Q1, Q3) (N hh)	138.0 (41.2, 385.3) (*n* = 27)	85.0 (48.9, 121.5) (*n* = 39)	95.0 (54.4, 131.7) (*n* = 210)	81.7 (43.8, 151.7) (*n* = 146)	91.0 (47.5, 142.4) (*n* = 422)
**Overall TSU**					
No TSU detected by SUMs or visual observation: N (%)	309 (77.2%)	359 (90.2%)	175 (44.4%)	224 (57.0%)	1068 (67.4%)
Any TSU detected by SUMs or visual observation: N (%)	91 (22.8%)	39 (9.8%)	219 (55.6%)	168 (42.7%)	517 (32.6%)

^1^ Percent of households with valid SUM data.

**Table 4:** Top reasons reported for traditional stove use among the intervention households during pregnancy.

IRC	Top Three Ranked Reasons for Traditional Stove Use (N Reporting; Percent of the Intervention Households)
Guatemala	Needing to cook large quantities of food for special occasions (32; 8.0%) Challenges with cleaning or maintaining the LPG stove (20; 5.0%) Preparing a traditional dish (14; 3.5%)
India	Unsure how to check for or respond to a leak (1; 0.3%) Challenges with cleaning or maintaining the LPG stove (1; 0.3%) General feeling of insecurity with the LPG stove (1, 0.3%) Other household members using the traditional stove (1; 0.3%)
Peru	Other household members using the traditional stove (72; 18.3%) Preparing a traditional dish (i.e., burning lamb’s head in the traditional stove) (33; 8.4%) Running out of LPG (28; 7.1%)
Rwanda	Running out of LPG (32; 8.1%) Other household members using the traditional stove (11; 2.8%) Concerns about the LPG cylinder exploding/burns (7; 1.8%)

**Table 5:** LPG stove use in the intervention households during pregnancy via SUMs.

IRC	Guatemala	India	Peru	Rwanda	Total
Households receiving intervention	400	398	394	393	1585
Households with SUMs on LPG stove ≥ 2 wks during gestation: N (%)	55 (13.8%)	110 (27.6%)	50 (12.7%)	29 (7.4%)	244 (15.4%)
Days of monitoring: median (Q1, Q3)	128.0 (119.0, 142.0)	127.0 (101.2, 148.8)	116.0 (48.2, 134.0)	112.0 (76.0, 130.0)	123.0 (97.8, 142.0)
Percent of stove-use-monitored days with LPG stove use detected: median (Q1, Q3)	99.0 (96.1, 100.0)	93.8 (84.9, 99.4)	99.3 (97.2, 100.0)	99.3 (94.7, 100.0)	98.3 (90.5, 100.0)
LPG stove cooking minutes per day of LPG stove use, among those with ≥ 1 day of LPG stove use in gestation: median (Q1, Q3) (N hh)	299.3 (223.4, 439.8) (*n* = 55)	197.0 (159.7, 248.8) (*n* = 110)	285.4 (226.4, 341.0) (*n* = 50)	231.2 (187.7, 292.3) (*n* = 29)	232.5 (181.3, 301.6) (*n* = 244)

## Data Availability

The data that support the findings of this article are publicly available at the following DOI: https://doi.org/10.15139/S3/9DDEAW.

## Supplementary Materials

The following are available online at https://www.mdpi.com/article/10.3390/ijerph182312592/s1, Table S1: Distributional statistics of derived ambient temperatures at each IRC, and the resultant primary thresholds selected for the standard and sensitive (LPG), Table S2: Stove Repairs during Pregnancy, Table S3. SUMs in Control Households During Pregnancy, Table S4. LPG Deliveries during gestation.
