# Supplementary material for: Fidelity and Adherence to a Liquefied Petroleum Gas Stove and Fuel Intervention during Gestation: The Multi-Country Household Air Pollution Intervention Network (HAPIN) Randomized Controlled Trial
Source: Int J Environ Res Public Health. 2021 Nov 29;18(23):12592. doi: 10.3390/ijerph182312592 (PMC8656791; doi:10.3390/ijerph182312592)
Supplement: Supplementary file 1 [file ijerph-18-12592-s001.zip › ijerph-1422425-supplementary.pdf]

SUPPLEMENTAL MATERIAL

SUMs Methods 2

Table S1: Distributional statistics of derived ambient temperatures at each IRC, and the resultant primary thresholds selected for the standard and sensitive (LPG) 2

Table S2: Stove Repairs during Pregnancy 3

Table S3. SUMs in Control Households During Pregnancy 4

LPG Delivery 5

Table S4. LPG Deliveries during gestation 5

References 6

SUMs Methods

To identify cooking events, we employed two different versions of the FireFinder algorithm (Wilson, Williams, and Pillarisetti 2020): a standard version for traditional stoves, and a more sensitive version for LPG stoves, as in (Piedrahita et al. 2020; Johnson et al. 2021). The algorithms used a primary threshold temperature and minimum event temperatures calculated from the estimated ambient temperature distributions in each country. A primary goal of the setting-specific thresholds was to clearly distinguish cooking events from natural diurnal variations in ambient temperature.

Country-specific ambient temperature distributions were estimated using the median daily temperatures across all SUMs installed on LPG stoves. We utilized data only from SUMs on LPG stoves because of their lower thermal mass, and generally shorter cooking durations when using LPG. We then defined the primary thresholds for each country using the median of these median daily temperatures plus 4 or 8 standard deviations of the daily median temperatures, for sensitive and standard algorithms, respectively. (Table S1). For the standard algorithms, the median temperature plus eight times the standard deviation of the median daily temperatures was used as the primary threshold while for the sensitive algorithm, four times the standard deviation of the medians was used. These values were selected after iterative revision to minimize false identification of stove usage events (as assessed by visual inspection of timeseries plots), while maintaining sensitivity for identification of lower temperature LPG usage events (Table S1). For both the standard and sensitive FireFinder algorithms, the minimum event duration was 5 minutes, and any events within 30 minutes of each other were grouped together into a single event.

Table S1: Distributional statistics of derived ambient temperatures at each IRC, and the resultant primary thresholds selected for the standard and sensitive (LPG)

**IRC**	**Median of daily median temperature (C)**	**SD of median daily temperature (C)**	**LPG cooking event primary threshold (Median + 4* SD)**	**Traditional stove cooking event primary threshold (Median + 8* SD)**
**Guatemala**	20.7	3.7	35.6	50.5
**India**	29.8	3.3	42.8	55.9
**Peru**	14.8	2.8	26.1	37.5
**Rwanda**	25.1	2.7	35.9	46.4

Table S2: Stove Repairs during Pregnancy

**Repairs to LPG stoves, tanks, and accessories**					
**IRC**	**Guatemala**	**India**	**Peru**	**Rwanda**	**Total**
**Repairs by household**					
Number of intervention households requiring repair: N (%)	44 (11%)	22 (5.5%)	56 (14.2%)	65 (16.5%)	187 (11.8%)
Number of repairs per household: Mean (SD)	1.2 (0.6)	1.0 (0.0)	1.1 (0.4)	1.1 (0.3)	1.1 (0.4)
Wait Time in Days for repair: Mean (SD)	0.2 (0.6)	0.2 (0.5)	0.4 (0.9)	0.5 (2.7)	0.4 (1.7)
**All repairs**					
Total repairs by IRC	55	22	64	72	213
Stove repairs: N (%)	43 (78.2%)	11 (50.0%)	45 (70.3%)	52 (72.2%)	151 (70.9%)
Gas tank repairs: N (%)	0 (0.0%)	5 (22.7%)	13 (20.3%)	7 (9.7%)	25 (11.7%)
Rubber Tubing Repairs: N (%)	0 (0.0%)	4 (18.2%)	3 (4.7%)	2 (2.8%)	9 (4.2%)
Other Repairs: N (%)	12 (21.8%)	5 (22.7%)	3 (4.7%)	11 (15.3%)	31 (14.6%)

Table S3. SUMs in Control Households During Pregnancy

IRC	Guatemala	India	Peru	Rwanda	Total
Enrolled households	400	399	402	404	**1605**
**Traditional stove SUMs**					
Households with SUMs on traditional stove >= 2 weeks during gestation: *N (%)*	58 (14.5%)	74 (18.5%)	38 (9.5%)	30 (7.4%)	**200 (12.5%)**
Days of Monitoring: *Median [Q1, Q3]*	120.5 [111.2, 129.8]	141.5 [101.0, 157.5]	118.5 [56.2, 134.8]	105.0 [82.0, 120.5]	**121.0 [93.8, 144.2]**
Percent of observation days with TSU: *Median [Q1, Q3]*	90.1 [18.1; 96.8]	69.7 (27.5); 76.9	53.1 (34.2); 42.0	54.1 (31.7); 50.8	**70.1 (30.8); 80.7**
Households with < 1 day with TSU per 30 days of observation: *N (% of those with valid SUMs)*	1 (1.7%)	2 (2.7%)	2 (5.3%)	2 (6.7%)	**7 (3.5%)**
Cooking minutes per day of stove use, among those with 1+ day of TSU in gestation*: Median [Q1, Q3] (N hh)*	379.4 [279.0, 511.6] (N = 57)	250.5 [184.9, 338.4] (N =72)	149.4 [102.3, 182.2] (N =38)	200.8 [139.6, 253.0] (N =29)	**238.6** [163.1, 350.4] (N = 196)
**LPG Stove SUMs**					
Households with SUMs on LPG stove during gestation: *N (%)*	0 (0.0%)	7 (1.8%)	4 (1.0%)	0 (0.0%)	**11 (0.7%)**
Households with SUMs on LPG stove > 2 wks during gestation: *N (%)*	0 (0.0%)	5 (1.3%)	4 (1.0%)	0 (0.0%)	**9 (0.6%)**
Percent of observation days with LPG stove use: *Median [Q1, Q3]*	NA	92.2 [77.3, 96.6]	68.3 [40.6, 85.9]	NA	**NA**
Cooking minutes per day of stove use, among those with 1+ day of LPG stove use in gestation: *Median [Q1, Q3] (N hh)*	NA	128.6 [108.4, 181.0] (N = 5)	108.4 [88.9, 144.1] (N =3)	NA	**NA**

LPG Delivery

Both the amount of LPG in a typical cylinder and the number of cylinder deliveries during the gestational period varied by IRC (Table S4). Households in India requested the fewest LPG deliveries: after the initial delivery of 2 full 14.2kg cylinders, only 350 (87.2%) intervention households in India requested an LPG refill during pregnancy, and among these the median [Q1, Q3] number of LPG cylinder refills provided during pregnancy was 2.0 [2.0, 3.0]. On the other end of the spectrum, all Guatemalan intervention households requested LPG refills during pregnancy and the median [Q1, Q3] number of refills over this period was 11.5 [8.0, 16.0] (cylinders containing 11.3kg LPG). Requested fuel deliveries in households in Peru and Rwanda were between these.

Table S4. LPG Deliveries during gestation

**IRC**	Guatemala	India	Peru	Rwanda	Total
Amount of LPG in typical full cylinder *(kg)*	11.3	14.2	10.0	15.0	NA
Intervention households with at least one LPG refill in gestation[1]: *N (%)*	400 (100%)	350 (87.9%)	393 (99.2%)	390 (99.0%)	1532 (96.4%)
Number of LPG cylinder deliveries per household (N): *Median [Q1, Q3]*	11.5 [8.0, 16.0]	2.0 [2.0, 3.0]	9.0 [7.0, 10.0]	4.0 [3.0, 5.0]	6.0 [3.0, 10.0]

[1]Households not accounted for here did not request or receive an LPG refill during the gestational period.

References

Johnson, Michael, Ricardo Piedrahita, Ajay Pillarisetti, Matthew Shupler, Diana Menya, Madeleine Rossanese, Samantha Delapeña, et al. 2021. "Modeling Approaches and Performance for Estimating Personal Exposure to Household Air Pollution: A Case Study in Kenya." *Indoor Air*, March, ina.12790. https://doi.org/10.1111/ina.12790.

Piedrahita, Ricardo, Michael Johnson, Kelsey R. Bilsback, Christian L'Orange, John K. Kodros, Sarah Rose Eilenberg, Agnes Naluwagga, et al. 2020. "Comparing Regional Stove-Usage Patterns and Using Those Patterns to Model Indoor Air Quality Impacts." *Indoor Air* n/a (n/a). https://doi.org/10.1111/ina.12645.

Wilson, Daniel Lawrence, Kendra N. Williams, and Ajay Pillarisetti. 2020. "An Integrated Sensor Data Logging, Survey, and Analytics Platform for Field Research and Its Application in HAPIN, a Multi-Center Household Energy Intervention Trial." *Sustainability* 12 (5): 1805. https://doi.org/10.3390/su12051805.
